# Impact of Erythropoietin in the management of Hypoxic Ischaemic Encephalopathy in resource-constrained settings: protocol for a randomized control trial

**DOI:** 10.1186/s12883-020-01751-y

**Published:** 2020-05-04

**Authors:** Beatrice Ezenwa, Chinyere Ezeaka, Iretiola Fajolu, Anne Ogbenna, Omodele Olowoyeye, Obiyo Nwaiwu, Zainab Opoola, Gbenga Olorunfemi

**Affiliations:** 1 Neonatology unit, Department of Paediatrics, College of Medicine University of Lagos, Lagos, Nigeria; 2 Department of Paediatrics, Lagos University Teaching Hospital, Lagos, Nigeria; 3 Department of Haematology & Blood transfusion, College of Medicine University of Lagos, Lagos, Nigeria; 4 Department of Radiodiagnosis, College of Medicine University of Lagos, Lagos, Nigeria; 5 Department of Pharmacology, Therapeutics &Toxicology, College of Medicine University of Lagos, Lagos, Nigeria; 6 Division of Epidemiology and Biostatistics, School of Public Health, University of Witwatersrand, Johannesburg, South Africa

**Keywords:** Perinatal asphyxia, Hypoxic-ischemic encephalopathy, EPO and neurodevelopment

## Abstract

**Background:**

Perinatal asphyxia, more appropriately known as hypoxic-ischemic encephalopathy (HIE), is a condition characterized by clinical and laboratory evidence of acute or sub-acute brain injury resulting from systemic hypoxemia and/or reduced cerebral blood flow. HIE is a common and devastating clinical condition in resource-poor countries with poor treatment outcome. This paper describes the protocol for an ongoing study that aims to evaluate the neuroprotective effects of Erythropoietin (EPO) as compared to routine care in the management of moderate to severe HIE among term infants.

**Methods:**

This study is a double-blind randomized controlled trial that will be conducted in the neonatal wards of the Lagos University Teaching Hospital (LUTH), Lagos, Nigeria, over a two-year period after ethical approvals and consents. One hundred and twenty-eight term newborns (≥ 37 weeks gestation) diagnosed with moderate/ severe HIE at admission will be allocated by randomization to receive either EPO or normal saline. All the participants will be offered standard care according to the unit protocol for HIE. Baseline investigations and close monitoring of the babies are done until discharge. Participants are followed up for 2 years to monitor their outcome (death or neurological development) using standard instruments.

**Discussion:**

Previous trials had shown that EPO confers neuroprotective benefits and improve neurological and behavioral outcome in infants with HIE both singly or as an adjuvant to therapeutic hypothermia. This study hypothesized that administering EPO to newborns with moderate /severe HIE can positively influence their clinical and neurological outcomes and will provide evidence to either support or disprove the usefulness of Erythropoietin as a sole agent in the treatment of HIE, especially in resource-limited environment with the highest burden of the disease.

**Trial registration:**

The study has been registered with the Pan African Clinical trials registry on the 2nd of December 2018, with registration number PACTR201812814507775.

## Background

Perinatal asphyxia (PA), more appropriately known as hypoxic-ischemic encephalopathy (HIE), is a condition characterized by clinical and laboratory evidence of acute or sub-acute brain injury resulting from systemic hypoxemia and/or reduced cerebral blood flow [1]. The insult to the brain can occur in-utero, intrapartum or postnatally. Hypoxic-Ischemic Encephalopathy is the commonest etiology for neonatal encephalopathies and accounts for 23% of all term newborn deaths worldwide [2]. Severe HIE occur in 0.1–0.3% of births in resource-rich countries [2, 3]. However, the incidence of HIE is greater in low and middle-income countries (LMICs) [4] occurring in nearly 35% of neonatal admissions in India while different hospital-based studies in Nigeria documented between 2.6 and 30% [5–8]. Furthermore, Ekure et al [9] reported that HIE accounted for nearly 39% of perinatal mortalities at a tertiary hospital in Lagos, South-Western Nigeria. HIE can be mild, moderate or severe. However, the majority (about 90%) of cases of HIE that present to hospitals in Nigeria were moderate to severe [10]. The morbidity and mortality from HIE increases with increasing severity [10].

Survivors of moderate/ severe HIE have a high risk of developing severe cognitive and/or motor impairment, seizure disorders, deafness, and vision loss [11, 12] Neurologic abnormality at discharge is a strong predictor of long-term neurodevelopmental delay [13]. Many biomarkers of neurological damage resulting from asphyxia have been identified and investigated [14, 15]. Biomarkers such as nucleated red blood cells (NRBC) has been demonstrated to be markedly elevated in asphyxiated newborns and can be useful in predicting adverse neurological outcomes [13–15]. Inflammatory cytokines such as Interleukin-6 (IL-6) have been shown to accumulate early in asphyxia [16, 17]. Chiesa et al. [18] reported that IL-6 concentrations were more than 300-folds in babies with HIE as compared to normal infants. Furthermore, Creatinine kinase BB (CK-BB) has been shown to predict HIE early as its rise occurs in the first hours after brain injury [19].

Therapeutic hypothermia (HT) has emerged as the gold standard for treating HIE [20] Thus if HT is commenced within 6 hours of injury, morbidity and mortality among infants with HIE can be significantly reduced [21–24]. HT is not readily available in most LMICs such as Nigeria. It is imperative that efforts should be geared towards early diagnosis and recognition of the sequelae of HIE, and improvement in the quality of life of survivors of HIE. In Nigeria, the treatment of HIE is largely symptomatic and supportive despite the availability of beneficial treatment modalities elsewhere. Indeed, parents of babies with HIE may not be able to afford the high cost of the therapies.

Recently, some drugs such as Erythropoietin (EPO) have been found to possess positive neuroprotective, neuro-regenerative and anti-inflammatory effects in asphyxiated newborns [25–31]. EPO, hitherto widely used for Anemia of prematurity, achieves its neuroprotective effects through its anti-excitotoxic, antioxidant and anti-apoptotic effects on neurons and oligodendrocytes [25–30, 32]. It also has anti-inflammatory effects on astrocytes and microglial cells [25–28, 31]. EPO prevents NO-induced cell death, protects neurons from glutamate toxicity, [33] and has demonstrable effects on neurogenesis, differentiation and repair after injury [29, 30, 34, 35]. Several studies on human and animal models demonstrated improvement in both short and long-term neurodevelopmental outcomes among asphyxiated babies that were exposed to EPO [33, 36, 37]. Even when EPO was commenced as late as 1-week post asphyxial insult in rodents, there was still beneficial effects in behavioral outcome, neuronal protection and regeneration [38]. The systematic review by Garg et al. [39] indicated that EPO confers long-term neurodevelopmental protection on neonates with moderate/severe HIE. None of the trials conducted on EPO among newborns, have shown any significant side effect despite the high doses employed for asphyxia [33, 40, 41].

Though EPO can be used synergistically with HT, [41] studies have shown good and beneficial effects when used as a single agent in the treatment of HIE [33, 42]. This is especially important in settings where HT is non-existent or when intervention is delayed due to late presentation. Despite the poor outcome of HIE in Nigeria, there is no local evidence supporting the introduction of EPO as a routine drug for the prevention of early and late neurological complications of perinatal asphyxia in the country. This study aims to evaluate the neuroprotective effects of EPO in comparison to routine care in the management of HIE in term infants.

### Null hypothesis

Parenterally administered high dose EPO in newborns with HIE does not influence immediate or long-term outcome.

### Specific objectives

1. To describe the spectrum of asphyxia morbidities at the Lagos University Teaching Hospital
2. To compare the neurological outcomes of babies with moderate/severe HIE that had adjunctive treatment with EPO as compared to routine care in the immediate neonatal period and at 6, 12, 18 and 24 months of age
3. To document common complications and adverse drug reactions in the treated infants

## Methods/ design

### Study design

This study is a double-blind randomized controlled parallel-group trial with one to one allocation ratio. The study is prospectively registered with the PACTR.

### Study setting

This study is being conducted in the neonatal wards and clinics of the Lagos University Teaching Hospital (LUTH), Lagos, Nigeria and is ongoing as at the time of this submission. LUTH is a tertiary care hospital with facilities for neonatal care. The LUTH neonatal ward is the largest in the country with a capacity to admit more than 80 babies at a time. It has inborn and out-born sections with equal capacity for admissions.

### Population and sample size

Table 1 shows the inclusion and exclusion criteria. All consecutively admitted term neonates with a diagnosis of severe perinatal asphyxia and presenting within 24 h of life in LUTH will be recruited for the study if they meet inclusion criteria.

**Table 1:** Participants’ Inclusion and exclusion criteria for treatment

1. Enrollment criteria will include:	
• All term newborns (≥ 37 weeks gestation) diagnosed of moderate/ severe HIE at admission and who are < 24 h of age.	
• Evidence of perinatal depression based on delayed cry at birth and at least one of the following:	
1. Apgar score ≤ 3 at 5 min.	
2. Need for resuscitation at 10 min.	
3. PH < 7.1 in cord or Base deficit ≥15 mmol/L.	
4. Moderate or severe encephalopathy according to Sarnat and Sarnat staging [43]. (Table 2)	
5. History of an adverse intrapartum sentinel event such as abruptio placenta, uterine rupture, fetal distress, etc	
2. Exclusion criteria:	
• Severe congenital anomalies	
• Preterm babies	
• Infants > 24 h of age at time of presentation /consent	
• Those in which consent could not be obtained	
• Evidence of sepsis (confirmed according to unit protocol)	

### Sample size and power calculation

There was no previous study in our environment describing the efficacy of EPO administration on the outcome of HIE. Furthermore, there is a paucity of recent published data on mortality and neurodevelopmental impairment from perinatal asphyxia in our environment. Mortality rate from asphyxia was reported to be between 31.1 and 62.8% in some hospitals in Nigeria [5, 9, 10, 44]. Since no study reported the prevalence of neurodevelopmental impairment of asphyxiated babies at 2 years of life, we expected that the prevalence of a combination of the outcome of “death and neurodevelopmental impairment” at 2 years of life among moderate/ severe HIE in a resource-poor environment such as Nigeria should be higher than the prevalence of death only (31.1–62.8%). Thus, we assumed that the prevalence of “death and neurodevelopmental impairment at 2 years of life” among moderate/ severe HIE without EPO treatment in Nigeria will be about 65%. Additionally, Zhu et al. [40] in Zhengzhou, China found that 24.6% (*n* = 18/73) of babies who had EPO either died or had neurodevelopmental impairment while 43.8% who had placebo died or had neurodevelopmental impairment, giving a relative risk of 0.62 (95% CI: 0.41–0.94). Similarly, the range of protective relative risk that was reported or assumed by previous studies was between 0.65 to 0.71 [41, 45]. Hence, we utilized the power calculator in Stata version 16 statistical software (Stata Corp, USA) to calculate the minimum sample size to be 128 babies based on the assumption that 65% of infants with moderate/ severe HIE may likely die or develop neurodevelopmental impairment without treatment; assuming a protective relative risk of 0.6, 80% power and 10% loss to follow -up.

We utilized the Sarnat and Sarnat staging [43] of moderate or severe HIE for the recruitment of eligible participants (Table 2).

**Table 2:** Sarnat and Sarnat staging [43] of moderate or severe HIE

Category	Moderate HIE	Severe HIE
1. Level of consciousness	Lethargy or obtunded	Stupor or Coma
2. Spontaneous activity	Decreased	Absent
3. Tone	Hypotonia	flaccidity
4. Posture	Distal flexion	Decerebrate state
5. Primitive reflexes		
a. Suck	Weak or biting	Absent
b. Moro reflex	Incomplete	Absent
6. Autonomic nervous system		
a. Pupils	Constricted	Deviated, dilated, or nonreactive to light
b. Heart rate	Bradycardia	Variable heart rate
c. Respirations	Periodic breathing	Apnoea
7. Seizures	Common, focal or multifocal	Absent or uncommon
8. EEG activity	Low voltage changing to seizure activity	Burst suppression to isoelectric

Presence of ≥1 of these signs in any 3 of the above 8 categories is required for diagnosis

### Risk of bias

This is a double-blind randomized control trial. Each participant is allocated into the intervention or control group by simple randomization using randomly generated number cards in sequentially numbered sealed opaque envelopes. The randomization list was created and generated online for the study by the Biostatistician based in South Africa. The outcome assessors (Paediatric Neurologist, Radiologist and the Biostatistician) are blinded to the group allocation during the study period. The managing physicians, however, are not blinded to the intervention drugs.

### Intervention and procedure

The approach to the management of HIE in our center is supportive care. Therapeutic hypothermia is not available. Supportive care includes maintaining normothermia, fluid, electrolyte and glucose balance, respiratory support with continuous positive airway pressure (CPAP) and oxygen, control of seizures and management of other complications as they arise [46]. During this study, participants randomized into EPO group, receives 1000 IU/kg of Erythropoietin intravenously statim on admission then daily for 5 days. This dose regimen was modified from the pharmacokinetic study by Wu et al. [37] Placebo/ control group receives 2 ml of 0.9% Saline statim on admission then daily for 5 days.

### Clinical monitoring

All the participants are offered standard care according to the unit protocol for HIE. Fluid and electrolyte balance are maintained, oxygen therapy and other respiratory support modes are provided as necessary. Blood pressure is monitored during drug administrations and subsequently through a non-invasive blood pressure monitoring system. Daily monitoring and assessments are conducted until the 8th day of life using the Thompson’s score [47]. A peak score above 15 predicts a bad outcome, whereas maintaining a score below 10 which becomes 0 at 1 week of age suggests a good prognosis. The following clinical course and baseline characteristics are recorded on a pre-designed proforma: the grade of HIE (moderate or severe), the type of respiratory support needed, the presence of seizures, the time to establishment of full oral feedings through sucking and neurologic examination at discharge; the age at commencement of intervention, place of delivery, sex, gestational age at delivery, birth weight, head circumference, mother’s age, parity and intrapartum events are all recorded.

### Laboratory markers

Laboratory assessments for E/U/Cr, Ca^2+^ and PO_4_^−^, Complete Blood count with NRBC count and NRBC count per 100 white blood cells (WBC) are done at admission using a venous sample. The ratio of NRBC/100WBC is performed by a haematologist. The levels of NRBCs per 100 white blood cells (WBCs) correlates with acute as well as chronic asphyxia and can be used as a reliable marker of perinatal asphyxia and neurological outcome [13, 15].

### Cranial ultrasonography

All participants undergo a cranial ultrasound within 72 h of life looking for structural anomalies and cerebral echo densities. The ventricles are evaluated for size and configurations, the brain parenchyma checked for echogenicity, and presence of cystic encephalomalacia. Any echogenicity within the brain parenchyma, enlargement of the ventricles or the presence of encephalomalacia are considered as abnormal. Cranial ultrasound was chosen for this study over other imaging modalities due to its point of care application, relative affordability and ready availability in our setting. It is more affordable than MRI and is non-ionizing, unlike CT scan. However, it is important to note that it may not be the best tool for assessing intracranial abnormalities in term infants, as the extra-axial structures may not be well appreciated [48]. The cranial ultrasound is repeated at discharge for all participants to enable comparison. The scans are performed by a dedicated sonologist who is blinded to the intervention groups.

### Outcome

The outcome measures are assessed at different periods (Table 3). Full neurological examination is performed at discharge and at specified intervals (6,12,18,24 months) until the age of 2 years to assess for neurodevelopmental impairments using the Malawi Developmental Assessment Tool (MDAT) which has been validated for African children [49]. Another assessment tool that has been validated for the neurological assessment of neonates is the Bayley Scales of Infant and Toddler Development 3rd Edition.

**Table 3:** Outcome measures at different time periods

Domain	Outcome measure	Stage of HIE	T_**1**_ Discharge	T_**2**_ 4 months	T_**2**_ 6 months	T_**3**_ 12 months	T_**4**_ 18 months	T_**5**_ 24 months
Survival	Survival or death	X	X	X	X	X	X	X
Neurologic exam at discharge	Neurologically normal or abnormal	X	X					
Cranial Ultrasonography	Cranial USS abnormality	X	X					
Neurologic examination at clinic visits	Malawi Developmental Assessment Tool (MDAT) scores			X	X	X	X	X
Laboratory levels of EPO, NRBC, and NRBC/100 WBC	Levels of NRBC and NRBC/100WBC in relation to outcome	X	X					
Adverse reactions to EPO	Safety of the medications based on the presence or absence of any adverse event		X	X	X	X	X	X
Gross Motor Function Classification System (GMFCS) assessment system	Those with cerebral palsy are further categorized based on features present on standardized neurologic examination using GMFCS			X	X	X	X	X

T_1−_ T_5 -_ The five time periods for assessment; *NRBC* Nucleated red blood cells, *GMFCS* Gross motor function classification system.

(Bayley III) assessment tool. However, MDAT tool was preferred for this study since it takes cognizance of the culturally relevant differences in the neurodevelopmental assessment of African children. The Paediatric neurologist that assesses the neurodevelopmental status of the children will be blinded to the treatment arms.

#### Primary outcome measures

- The primary outcome is a composite outcome of death or neurodevelopmental impairment at 18–24 months of life. Neurodevelopmental impairment (NDI) is defined as the presence of any one of the following: hypertonia or spasticity, epilepsy, delayed milestones, cerebral palsy, Gross Motor Function Classification System (GMFCS) ≥2, MDAT *Z*-score for age ≤ 1.64 or scores less than expected for age using the MDAT chart [49].

#### Secondary outcome measures

- Survival at discharge and time to discharge. Those that survived to discharge is assessed as: neurologically normal (those who had normal tone and posture, were free from seizures, has established full feed by sucking and have normal neonatal reflexes – rooting, suck, grasp, tonic neck reflex, Moro and stepping reflexes) while neurologically abnormal are those with an abnormal tone and posture, poor suck or any abnormal neonatal reflexes at discharge)
- Association between the levels of NRBC and NRBC/100WBC with neonatal outcome
- Cranial USS evidence of ventricular enlargement, parenchymal echogenicity or cystic encephalomalacia.
- Safety of the medications based on the presence or absence of any adverse event such as:
  Hypertension: systolic blood pressure in a neonate which is above 95th percentile for age and sex on three separate occasions;
  Hypotension: a systolic blood pressure in a neonate which is below the 10th percentile for age and sex on three separate occasions
  Apnoiec attacks
  Thrombotic events;
  Polycythemia: venous hematocrit of greater than 65%.
- Any other adverse effect noted during the hospital stay or follow up.
- Those with cerebral palsy are further categorized based on features present on standardized neurologic examination, and classified as mild, moderate, or severe, with hemiplegia or diplegia, using the Gross Motor Function Classification System (GMFCS) assessment system.

### Data and safety monitoring plan

Study data are monitored by the Paediatric Pharmacologist and the Biostatistician who are not involved with the day to day management of the recruited patients. An interim analysis for neonatal outcome is scheduled after 6 months of commencement of study in the absence of documented serious adverse effects. A decision to terminate the trial will be taken by the Paediatric Pharmacologist and the principal investigator.

### Serious adverse events

A serious adverse event for this study is defined as any life-threatening events such as thrombotic events, severe hypertension or hypotension leading to death experienced by the infant following drug administrations or any other adverse event as previously described by other researchers [45]. All adverse events, both serious and non-serious, expected or unexpected, are recorded during the clinical trial and will be transmitted by the investigators to the designated competent authorities following hospital guidelines.

### Statistical analysis

Statistical analysis will be performed using Stata version 16.0 (Stata Corp, USA) statistical software after importing the data from the Excel spreadsheet. Descriptive statistics will be computed for socio-demographic characteristics and expressed as frequency tables and charts. Continuous variables that are normally distributed will be expressed as mean ± standard deviation (SD) while non-normally distributed continuous variables will be expressed as median and interquartile range (IQR). Association between categories of HIE and NRBC/100WBC will be tested using Students t-test. Also, the association between the grades of HIE and the degree of neurodevelopmental impairment will be conducted using the Pearson’s Chi-square. Pearson’s Chi-square will be used to compare the Gross motor function among the categories of HIE. Time to discharge, time to death and time to achieving major neurological milestones will be evaluated using the survival analysis techniques. Kaplan Meir’s plot and Cox proportional hazard regression modelling will be conducted with severity (moderate or severe) of HIE as a primary explanatory variable. At the end-point, after 2 years of follow-up, the association between intervention arm and the end point parameters will be assessed using univariable and multivariable logistic regression modelling. Two-tailed test of hypothesis will be assumed and a *P*-value < 0.05 will be taken as statistically significant level.

## Discussions

HIE is prevalent in resource-poor countries. The absence of facilities for therapeutic hypothermia reduces the chances for survival and increases the risk of morbidity. The use of EPO has been shown by several trials conducted in newborn infants with HIE to be beneficial in reducing morbidity and improve neurodevelopmental outcome even in infants that did not receive therapeutic hypothermia [40]. The previous studies did not document significant adverse reactions with the use of the drug. The systematic review by Garg et al [39] concluded that EPO improves neurodevelopmental outcome and is quite safe in neonates with hypoxic-ischaemic encephalopathy. More evidence to strengthen these findings will enhance its continued use. This study is expected to provide further evidence to either support or disprove the usefulness of Erythropoietin as a sole agent in the treatment of HIE. Either way, it will aid decision making and counselling of parents by newborn care providers treating infants with HIE, especially in resource-limited environment. Moreover, evidence-based, cost-effective measures that will not only curtail mortality but will also enhance the quality of life of survivors of HIE should be encouraged.

## Data Availability

Data sharing does not apply to this article as no datasets were generated or analysed during the current study.

## Abbreviations

PA: Perinatal asphyxia
HIE: Hypoxic ischaemic encephalopathy
EPO: Erythropoietin
LUTH: Lagos University Teaching Hospital
LMIC: Low and middle-income countries
NRBC: Nucleated red blood cells
CK-BB: Brain Creatinine kinase
IL: Interleukin
HT: Therapeutic Hypothermia
CPAP: Continuous positive airway pressure
WBC: White blood cell
MDAT: Malawi Developmental Assessment Tool
USS: Ultrasound
GMFCS: Gross Motor Function Classification System
WHO: World Health Organization
GCP: Good Clinical Practice
IQR: Interquartile range
HREC: Health research and ethics committee
